# The Adjuvant Activity of Alphavirus Replicons Is Enhanced by Incorporating the Microbial Molecule Flagellin into the Replicon

**DOI:** 10.1371/journal.pone.0065964

**Published:** 2013-06-13

**Authors:** Maria L. Knudsen, Daniel X. Johansson, Linda Kostic, Eva K. L. Nordström, Karin Tegerstedt, Anna Pasetto, Steven E. Applequist, Karl Ljungberg, Jean-Claude Sirard, Peter Liljeström

**Affiliations:** 1 Department of Microbiology, Tumor and Cell Biology, Karolinska Institutet, Stockholm, Sweden; 2 Unit of Immunization, Swedish Institute for Communicable Disease Control, Solna, Sweden; 3 Department of Medicine, Center for Infectious Medicine, F59, Karolinska Institutet, Karolinska University Hospital at Huddinge, Stockholm, Sweden; 4 Institut Pasteur de Lille, Center for Infection and Immunity of Lille, Lille, France; 5 Institut National de la Santé et de la Recherche Médicale, Lille, France; 6 Centre National de la Recherche Scientifique, Lille, France; 7 Univ Lille Nord de France, Lille, France; Federal University of São Paulo, Brazil

## Abstract

Ligands of pattern recognition receptors (PRRs) including Toll-like receptors (TLRs) stimulate innate and adaptive immune responses and are considered as potent adjuvants. Combinations of ligands might act in synergy to induce stronger and broader immune responses compared to stand-alone ligands. Alphaviruses stimulate endosomal TLRs 3, 7 and 8 as well as the cytoplasmic PRR MDA-5, resulting in induction of a strong type I interferon (IFN) response. Bacterial flagellin stimulates TLR5 and when delivered intracellularly the cytosolic PRR NLRC4, leading to secretion of proinflammatory cytokines. Both alphaviruses and flagellin have independently been shown to act as adjuvants for antigen-specific antibody responses. Here, we hypothesized that alphavirus and flagellin would act in synergy when combined. We therefore cloned the *Salmonella* Typhimurium flagellin (FliC) gene into an alphavirus replicon and assessed its adjuvant activity on the antibody response against co-administered antigen. In mice immunized with recombinant alphavirus, antibody responses were greatly enhanced compared to soluble FliC or control alphavirus. Both IgG1 and IgG2a/c responses were increased, indicating an enhancement of both Th1 and Th2 type responses. The adjuvant activity of FliC-expressing alphavirus was diminished but not abolished in the absence of TLR5 or type I IFN signaling, suggesting the contribution of several signaling pathways and some synergistic and redundant activity of its components. Thus, we have created a recombinant adjuvant that stimulates multiple signaling pathways of innate immunity resulting in a strong and broad antibody response.

## Introduction

Vaccines based on live-attenuated viruses are effective in inducing antibody responses; however, this approach is not feasible for viruses such as HIV-1 due to safety concerns. Many vaccines are composed of purified protein antigens that are safe and immunogenic but intrinsically not able to trigger an effective antibody response due to the absence of danger signals. Such vaccines are therefore formulated with an adjuvant to increase the magnitude of immune responses. Adjuvants also shape the immune response by modulating the balance between Th1 and Th2 responses [1]. The vaccines and adjuvants used today were largely developed by empirical approaches, and their modes of action are mostly not well characterized. Recently, the capability to stimulate innate immune responses through pattern recognition receptors (PRRs) was associated with vaccine potency to promote specific adaptive immune responses. For example, development of B cell responses is highly dependent on signaling through Toll-like receptors (TLRs) [2]. Also, one of the most successful vaccines ever made, the live-attenuated yellow fever vaccine, induces type I interferons (IFNs) and activates dendritic cells through multiple PRRs [3], [4]. Several studies suggest that combinations of agonists of different TLRs may further increase adaptive immune responses in a synergistic manner [5], [6], [7], [8], [9]. This knowledge has led to the pursuit of adjuvants that stimulate receptors of innate immunity.

Flagellin is the main component of the bacterial flagellum found on bacteria and is detected by TLR5 on cell surfaces [10] and by NLRC4 in the cytoplasm [11], [12]. Dendritic cells are activated and matured by flagellin administered in its soluble form [13] or expressed from a viral vector, as has been demonstrated with paramyxovirus simian virus 5 [14], adenovirus [15] and vesicular stomatitis virus [16]. Due to these properties, flagellin has been investigated for use as an adjuvant and has been shown to induce enhanced antigen-specific antibody responses as well as CD4+ and CD8+ T cell responses in animal models [17], [18], [19], [20], [21], [22], [23], [24], [25], [26], [27]. In most vaccination models, the adjuvant activity of flagellin was associated with TLR5 signaling [19], [26], [28]. Flagellin has been tested in clinical trials as a protein fused with an influenza antigen, demonstrating that flagellin is safe and well-tolerated in humans and functions as an adjuvant for the induction of neutralizing antibodies [29], [30], [31], [32]. The flagellin adjuvant has also been tested as a DNA plasmid [20] and has been incorporated into virus-like particles (VLPs) with HIV or influenza protein antigens, leading to enhanced antigen-specific antibody responses [21], [33]. Soluble flagellin promotes Th2 type responses [22], [34] whereas flagellin incorporated in VLPs activates a Th1 response [21].

Alphavirus replicons are essentially alphaviruses in which the genes encoding the structural proteins have been replaced with a transgene of interest. Alphavirus replicons possess adjuvant properties in that their RNA is self-amplifying due to the presence of the genes encoding the alphavirus replicase. RNA amplification occurs in the cytoplasm and results in the production of RNA intermediates that can stimulate PRRs including endosomal TLR3 [35], TLR7 and TLR8 [36]. Cytoplasmic PRRs such as melanoma differentiation-associated gene 5 (MDA-5) [37] and Protein Kinase RNA-activated (PKR) [38], [39] are also activated by alphaviral RNA. The signaling through PRRs results in the production of large amounts of type I IFNs [40], programmed cell death [41], and induction of antigen-specific adaptive immune responses [5], [6]. It has previously been demonstrated that administration of alphavirus replicon particles with protein antigen into mice results in enhanced antibody responses specific for the antigen [43], [44]. Vaccination with alphavirus replicons triggers a Th1-biased response that is highly dependent on type I IFN signaling [43].

Here, we hypothesized that incorporating flagellin into an alphavirus replicon would increase antigen-specific antibody responses. We therefore constructed Semliki Forest virus (SFV) replicon particles (VREP) that encode flagellin in the RNA genome. The recombinant flagellin-expressing virus was then co-administered with a model antigen and compared to control virus. Vaccination with recombinant virus was found to significantly enhance antigen-specific antibody responses compared to vaccination based on soluble flagellin protein or control VREP. Analysis of the antibody isotype profile indicated that the recombinant replicon induced both Th1 and Th2 type immunity. In the absence of either type I IFN or TLR5 signaling, the immune response was diminished but not completely abolished, demonstrating that the adjuvant activity of the recombinant replicon depends on several immune-potentiating pathways.

## Materials and Methods

### Proteins

β-galactosidase (β-Gal; Roche) and ovalbumin (OVA; Calbiochem, Merck) used for mouse immunizations and ELISA. A Limulus Amebocyte Lysate test (performed by the Swedish Institute for Communicable Disease Control, Stockholm, Sweden) showed that endotoxin levels were <0.05 EU/µg.

The native/wild type (WT) soluble FliC flagellin (sFliC-WT) was obtained from *Salmonella enterica* serovar Typhimurium as described previously [23]. The recombinant sFliC-D3 that contains a deletion in amino acids 174–400 was also used since it has been shown to be intrinsically poorly immunogenic [23].

### Viruses

Single-round infectious VREP encoding FliC-WT (VREP-FliC-WT) or FliC-D3 (VREP-FliC-D3) were constructed by first amplifying the FliC-WT and FliC-D3 genes from the pBS-FliC-WT and pBS-FliC-D3 plasmids (described in [23]), respectively, with primers with *Bam*HI and *Spe*I overhangs as well as a Kozak sequence. The FliC-WT and FliC-D3 PCR products were then cloned into the VREP vector. Plasmids were prepared using the EndoFree Plasmid Maxi kit (Qiagen, Hilden, Germany), and viruses were produced using the VREP two-helper RNA system as previously described [45]. VREP encoding β-Gal (VREP-LacZ), ovalbumin (OVA) (VREP-OVA) or enhanced green fluorescent protein (EGFP) (VREP-EGFP) were constructed as previously described [45]. Viral stock titers were determined using standard immunofluorescence methods [46].

**Figure 1:**
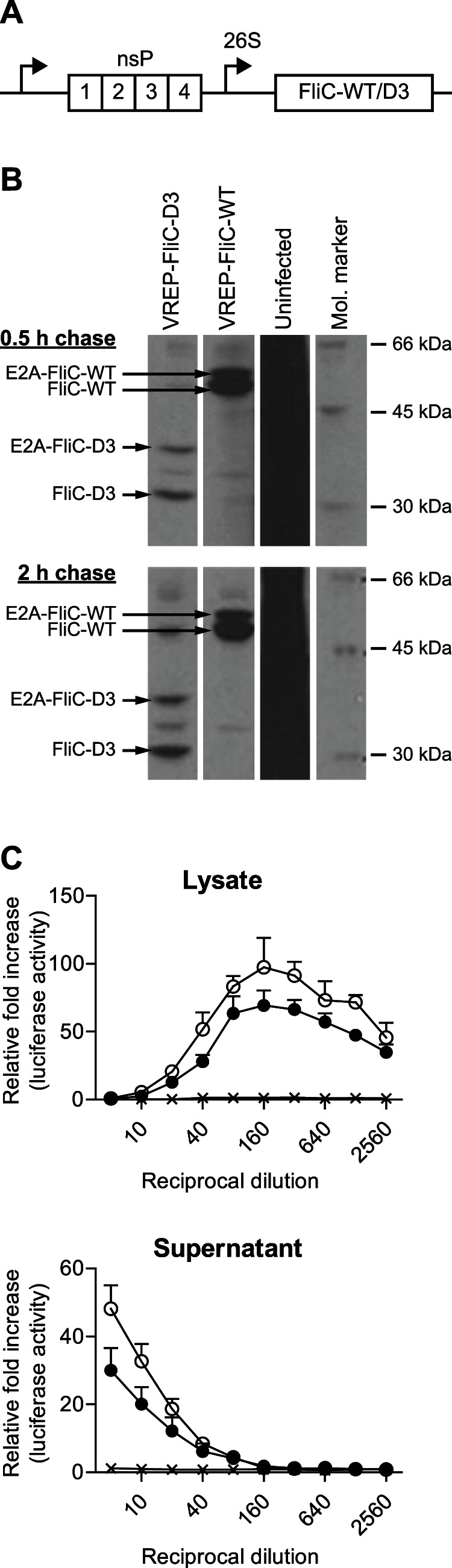
Constructs and TLR5-specific bioactivity. (A) Schematic illustration of VREP-FliC-WT/D3. nsP = non-structural proteins (replicase); 26S = subgenomic 26S promoter. (B) Stability of flagellin expressed from VREP. Cells were infected with VREP-FliC-D3, VREP-FliC-WT or left uninfected. Cells were then pulsed with ^35^S-methionine and chased for 0.5 h and 2 h. Total lysate proteins (non-immunoprecipitated) were separated by SDS-PAGE and detected by autoradiography. Distinct protein bands are visible in lysates from cells infected with VREP-FliC-D3 or VREP-FliC-WT due to translational shut-off of host proteins induced by VREP. In contrast, lysates from uninfected cells are seen as a black smear due to expression of many proteins. E2A is a translational enhancer (see Materials and Methods for description). (C) TLR5 bioassay. BHK-21 cells were infected with VREP-FliC-D3 (open circle), VREP-FliC-WT (filled circle), VREP-LacZ (X) or left uninfected. Supernatants and lysates from infected BHK-21 cells were then analyzed for their signaling through TLR5 in Caco-Rumbo cells, assayed in quadruplicates. Results are expressed as fold induction of luminescence in Caco-Rumbo cells incubated with supernatant or lysate from infected cells compared to incubation with supernatant or lysate from uninfected cells.

VREP-OVA, VREP-FliC-WT and VREP-FliC-D3 contain a translational enhancer (E2A) inserted directly upstream of the transgene. E2A consists of the first 34 amino acids of the SFV capsid gene, which contains a translational enhancer [47], and the 17 amino acid long 2A from foot-and-mouth disease virus, which promotes ribosomal ‘skipping’ during translation [48], [49]. In this process, the nascent 2A peptide modifies the activity of the ribosome so that the ester linkage between tRNA and the C-terminal amino acid of 2A is hydrolyzed, resulting in release of the nascent E2A peptide from the ribosome. The ribosome then continues translating the downstream sequence, thereby producing a new peptide that is not attached to E2A. In some cases, however, incomplete ribosomal skipping occurs, and a non-cleaved E2A-transgene peptide is produced, as illustrated in Fig. 1B.

### Metabolic Labeling (Pulse-chase)

BHK-21 cells were cultured in complete BHK-21 medium (Glasgow minimal essential medium BHK-21 supplemented with 5% fetal calf serum, 10% tryptose phosphate broth, 10 mM Hepes, 2 mM L-glutamine, 0.1 U/ml penicillin, 0.1 µg/ml streptomycin (Gibco, Invitrogen)). Cells were infected with VREP-FliC-WT or VREP-FliC-D3 at a multiplicity of infection (MOI) 20 and incubated overnight, then starved for 30 min with MEM lacking methionine (MP Biomedicals) supplemented with 2 mM L-glutamine, 10 mM Hepes and pulsed for 10 min with the same medium supplemented with 100 µCi/ml ^35^S-methionine. Cells were then washed and incubated with chase medium (Earle’s minimal essential medium supplemented with 2 mM L-glutamine, 10 mM Hepes and 150 µg/ml unlabeled methionine) for 0.5 or 2 h. Lysis buffer (1% SDS, 50 mM NaCitrate pH 6.0) was then added, and total cell lysate was collected and centrifuged to remove cellular debris. The lysate was then boiled for 5 min and run on an SDS-gel. A rainbow [^14^C] methylated protein molecular weight marker (Amersham, GE Healthcare) was run on the gel as reference. The gel was then soaked in a 1 M sodium salicylate (Merck) bath for 20 minutes and dried in a gel dryer. Radioactive proteins were detected on an X-ray film (Fujifilm).

### TLR5 Bioassay

BHK-21 cells were infected with VREP-FliC-WT, VREP-FliC-D3 or VREP-LacZ at MOI 20 and incubated overnight in 2 ml complete BHK-21 medium. Medium was then collected, and cell lysates were obtained by adding 300 µl of 100°C PBS with protease inhibitors (1 µg/ml phenylmethylsulfonyl fluoride and 10 mM 2-iodoacetamide). Detachment of cells was achieved with a cell scraper. Lysates were incubated at 100°C for 5 min to complete lysing of cells. Medium and cell lysates were then analyzed on the Caco-Rumbo cell line. These cells were previously developed by co-transfecting the Caco-2 human colon adenocarcinoma cell line with a plasmid encoding TLR5 and a plasmid encoding a luciferase gene under the control of the human *CCL20* promoter [50]. Samples were tested on the Caco-Rumbo cell line as described previously [23]. Results are expressed as the ratio of luminescence between Caco-Rumbo cells incubated with or without material from infected cells.

### Mice and Immunizations

129sv/ew, BALB/c, C57BL/6, *Ifnar1^−/−^* (129sv/ew background) and *Tlr5^−/−^* (C57BL/6 background) mice were bred at the animal facility at the Department of Microbiology, Tumor and Cell Biology at Karolinska Institutet, Sweden. *Tlr5^−/−^* mice were kindly provided by Professor Shizuo Akira, Osaka University, Japan [51]. C57BL/6N mice were purchased from Charles River (Germany). Mice were 6–12 weeks old at the initiation of experiments and were age and sex-matched within each experiment. Each vaccinated group within an experiment consisted of five to six mice and one to four control mice. All experiments were performed in at least two replications. All mice were kept at the Department of Microbiology, Tumor and Cell Biology at Karolinska Institutet, Sweden in accordance with the recommendations of the National Board for Laboratory Animals. The protocol was approved by the local ethics committee, Stockholms norra djurförsöksetiska nämnd, Permit Number N191/11.

Mice were immunized subcutaneously with 200 µl of PBS containing one or more of the following: β-Gal (10 µg), OVA (15 µg), sFliC-D3, sFliC-WT, VREP-OVA, VREP-LacZ, VREP-FliC-WT or VREP-FliC-D3. Specific doses of adjuvants are indicated in the figure legends. Blood was collected 3 weeks post-immunization.

### ELISA

ELISA plates (Immunosorp, Nunc, Denmark) were coated overnight with 1 µg/ml β-Gal, 10 µg/ml OVA or 5 µg/ml sFliC-WT diluted in 0.1 M carbonate buffer at 4°C. After washing the plates three times with PBS plus 0.05% Tween, plates were blocked with PBS plus 5% skim milk for 1 h at room temperature. Serum was then serially diluted in PBS containing 0.05% Tween and 5% skim milk. After 2 h incubation at room temperature, plates were washed five times with PBS plus 0.05% Tween, and horseradish peroxidase-conjugated anti-mouse-IgG, anti-mouse-IgG1, anti-mouse-IgG2a or anti-mouse-IgG2c (all Southern Biotech, Birmingham, AL) was added and incubated for 1.5 h. Plates were subsequently washed five times with PBS plus 0.05% Tween and the o-phenylenediamine dihydrochloride substrate (Sigma) was added for detection of antibodies. The reaction was stopped after 15 min with 1 M HCl, and the optical density (OD) at 490 nm was read using an ELISA reader. For calculation of endpoint titers, a cutoff value of OD 0.3 was used. Results are expressed as group means+SEM.

### Statistics

Statistical analyses were performed using the GraphPad Prism 5 software (GraphPad Software Inc., La Jolla, CA). A *P*-value of <0.05 was considered statistically significant.

## Results

### Flagellin Expressed from VREP Signals through TLR5

In this study, we constructed SFV-based replicon particles (VREP) encoding flagellin from *S.* Typhimurium. To this aim, two types of flagellin were used: the native form FliC-WT and the recombinant FliC-D3 that contains a deletion in the hyperimmunogenic region of flagellin (amino acids 174–400). Thus, FliC-D3 has reduced intrinsic immunogenicity, thereby preventing antibody responses specific for flagellin without compromising innate immune signaling [23], [52]. FliC-WT and FliC-D3 were cloned into VREP to form VREP-FliC-WT and VREP-FliC-D3, respectively (Fig. 1A). Since VREP does not contain the genes encoding the structural proteins of SFV, infection is non-productive and no new viruses are formed after infection.

After cell infection with the recombinant virus particles, flagellin is expected to be expressed intracellularly. First we analyzed whether flagellin is produced following infection of BHK-21 cells with VREP-FliC-WT or VREP-FliC-D3. We confirmed that both VREP-FliC-WT and VREP-FliC-D3 promote intracellular expression of flagellin as determined by western blot analysis of cell lysates (data not shown). Stability of flagellin production was analyzed after pulsing with ^35^S-methionine and chasing for 0.5 h and 2 h. This experiment showed that both FliC-WT and FliC-D3 remained in cells for 2 h (Fig. 1B).

We then assessed whether flagellin expressed from VREP-infected cells could signal through TLR5, and whether activity of flagellin is restricted to the intracellular or extracellular compartment when released. We therefore infected BHK-21 cells with VREP-FliC-WT, VREP-FliC-D3 or VREP-LacZ as control and collected cell lysates and culture supernatants for analysis in a TLR5 bioassay. Using Caco-Rumbo cells, which are human colon epithelial cells that have been co-transfected with plasmids encoding TLR5 and luciferase under transcriptional control of a CCL20-inducible promoter [50], we demonstrated that cell lysates and to a lesser degree supernatants isolated from flagellin-expressing VREP were able to activate TLR5 signaling in contrast to samples isolated from control VREP (Fig. 1C). It is noteworthy that TLR5-stimulating activity was decreased with higher amounts of cell lysates, likely due to toxicity or inhibitory factors of concentrated samples containing protease inhibitors. In conclusion, we demonstrated that infection with VREP-FliC-WT or VREP-FliC-D3 results in production of flagellin capable of signaling through TLR5.

### Flagellin Expressed from VREP does not Enhance the Antibody Response Against Antigen Encoded by VREP

**Figure 2:**
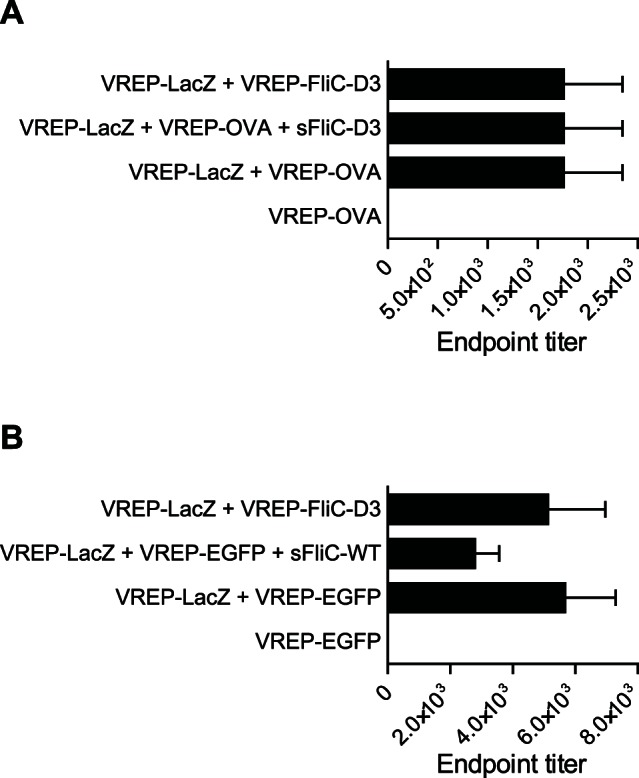
Antibody responses against β-Gal expressed from VREP. 129sv/ew (A) or BALB/c (B) mice were immunized with the indicated regimen. Doses used were: 10^6^ infectious units (IU) of VREP particles, 0.2 µg sFliC-D3 and 1 µg sFliC-WT. When two different VREP particles were given to the same mouse, 5×10^5^ IU of each VREP was given. Each immunized group consisted of five mice, and one to two control mice was used. Serum was assayed for anti-β-Gal IgG by ELISA. A one-way ANOVA with Bonferroni post-hoc test of the response between vaccinated groups revealed no significant differences.

Antigens expressed from VREP have previously been shown to be capable of inducing strong antigen-specific antibody responses [43], [53], [54]. In addition, flagellin administered either as protein or encoded by DNA has been shown to have an adjuvant effect on the humoral response [17], [18], [19], [20], [21], [22], [23]. Since FliC and VREP stimulate different PRRs, we asked whether responses against antigen expressed from VREP could be further enhanced by flagellin, either administered in its soluble form or expressed from VREP. We therefore immunized mice with VREP-LacZ as the antigen-expressing VREP, mixed with VREP-FliC-D3, with VREP-OVA, or with VREP-OVA and soluble flagellin. Assessing the β-Gal-specific IgG response, we observed no significant differences between the groups (Fig. 2). Thus, flagellin in its soluble form or expressed from VREP does not act as an adjuvant for IgG responses against VREP-encoded antigen.

### Incorporating the Flagellin Gene into VREP Enhances the Adjuvant Effect on Antibody Responses

It has previously been demonstrated that VREP particles have an adjuvant effect on the antigen-specific IgG response when coimmunized with protein antigen [43], [44]. We hypothesized that we could achieve an additional adjuvant effect by combining VREP and flagellin as an adjuvant for protein antigen. For this purpose, we immunized mice with β-Gal alone or in combination with 10^4^, 10^5^ or 10^6^ infectious units (IU) of VREP encoding either FliC-D3 or control VREP expressing ovalbumin (OVA). Total IgG responses as well as IgG1 and IgG2a antibodies targeted at β-Gal were then assessed with an ELISA assay.

**Figure 3:**
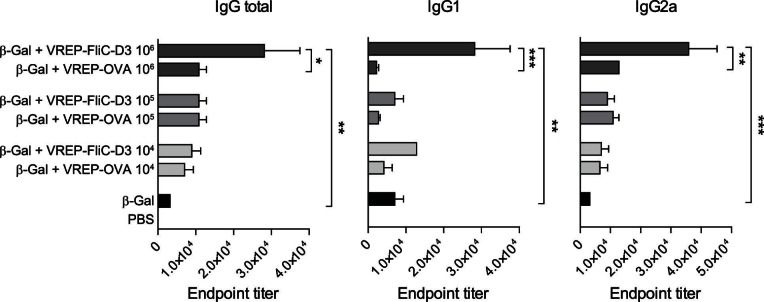
Antibody responses induced by different doses of VREP-FliC-D3. 129sv/ew mice were immunized with β-Gal alone or with adjuvant, as indicated. Three different doses of VREP-FliC-D3 and VREP-OVA were used: 10^6^ IU, 10^5^ IU and 10^4^ IU. Control mice were given PBS. Each immunized group consisted of five mice, and four control mice were used. Serum was assayed for anti-β-Gal IgG, IgG1 and IgG2a by ELISA. A one-way ANOVA with Bonferroni post-hoc test was used to compare the response between mice given the same dose of adjuvant as well as between groups given adjuvant and the control group given β-Gal without adjuvant. *P<0.05, **P<0.01 and ***P<0.001.

In accordance with previous results, we observed an increased IgG response against β-Gal when β-Gal was co-immunized with VREP-OVA (Fig. 3). The response was similar in magnitude at all three doses tested and was mainly characterized by IgG2a antibodies, indicating a Th1 type response. For VREP-FliC-D3, an adjuvant effect was also observed. At 10^6^ IU, the adjuvant effect increased to a level stronger than an equivalent dose of VREP-OVA. This response was evident both in the IgG1 and IgG2a responses, indicating that VREP-FliC-D3 has an adjuvant effect on both the Th2 and Th1 type responses. Based on these results, we selected a dose of 10^6^ IU for further studies.

**Figure 4:**
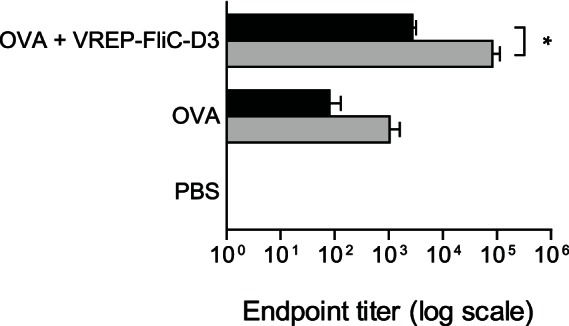
Antibody responses after multiple immunizations. C57/BL6 mice were immunized two times with OVA protein alone or mixed with 10^6^ IU VREP-FliC-D3. Control mice were given PBS. Each immunized group consisted of five mice, and two control mice were used. Serum was assayed for anti-OVA IgG by ELISA after one (black bars) or two (gray bars) immunizations. A Student’s *t*-test was used to compare the response after one and two administrations of the same vaccine. * *P*<0.05.

To ask whether the response induced by VREP-FliC-D3 could be further increased by multiple immunizations, we immunized C57BL/6 mice with OVA alone or together with VREP-FliC-D3 up to two times and assessed the antibody response against OVA. Compared to immunization with OVA alone, the response was increased 34-fold when OVA was co-administered with VREP-FliC-D3. This response was further increased 30-fold by a second immunization of OVA mixed with VREP-FliC-D3, demonstrating that the VREP-FliC-D3 adjuvant is suitable for use in multiple administrations in prime-boost regimens (Fig. 4). In conclusion, our data indicated that VREP expressing intracellular flagellin is a more potent adjuvant than VREP alone.

### Soluble Flagellin and VREP do not Act in Synergy

**Figure 5:**
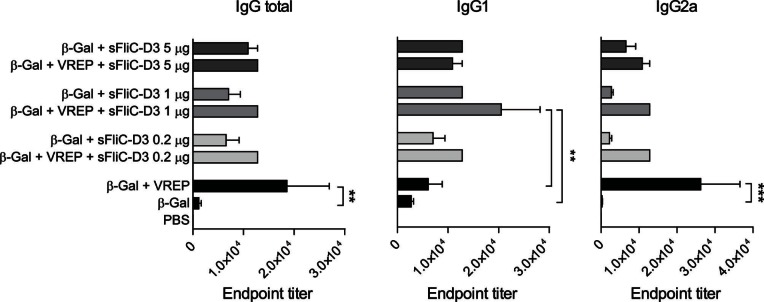
Antibody responses induced by VREP and sFliC-D3. 129sv/ew mice were immunized with β-Gal alone or with indicated adjuvant. VREP-OVA is indicated as ‘VREP’, and the dose used was 10^6^ IU. Doses of sFliC-D3 are indicated in the figure. Control mice were given PBS. Each immunized group consisted of five mice, and two control mice were used. Serum was assayed for anti-β-Gal IgG, IgG1 and IgG2a by ELISA. A one-way ANOVA with Bonferroni post-hoc test was used to compare the response between mice given the same dose of sFliC-D3, between mice given VREP+sFliC-D3 and mice given VREP, as well as all adjuvanted groups and mice given β-Gal alone. **P<0.01 and ***P<0.001.

We next investigated whether the adjuvant effect of VREP-FliC-D3 was dependent on intracellular expression of flagellin. For this purpose, we hypothesized that antibody responses would differ when co-immunizing control VREP-OVA and soluble flagellin FliC-D3 (sFliC-D3) compared to control VREP-OVA or sFliC-D3 alone. We therefore immunized mice with β-Gal antigen alone or together with soluble sFliC-D3 or VREP-OVA and sFliC-D3. Previous results have shown that there is no difference in the antigen-specific IgG response promoted by flagellin at doses ranging from 30 µg down to 0.1 µg [22]. Here, we tested three different doses of sFliC-D3: 0.2, 1 and 5 µg. Analyzing the IgG response with ELISA, we observed that co-immunizing VREP with soluble flagellin did not result in an added adjuvant effect, compared to using sFliC-D3 or VREP alone (Fig. 5). In fact, adding sFliC-D3 in the vaccine appeared to inhibit the effect of VREP on total IgG and IgG2a responses, although this difference was not statistically significant. Also, in accordance with previous results, there were no significant differences in the response induced by the three doses of sFliC-D3. Based on these results, we selected a dose of 0.2 µg of flagellin, which was the lowest dose tested, for further studies. In conclusion, our data indicated that VREP enhanced the adjuvant effect of soluble flagellin, although soluble flagellin did not enhance the adjuvant effect of VREP.

### Both WT and Truncated Flagellin Expressed from VREP Exert Adjuvant Activities

**Figure 6:**
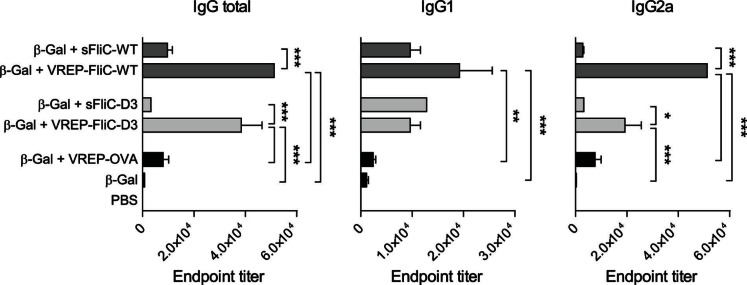
Adjuvant effect of VREP encoding FliC-WT or FliC-D3. 129sv/ew mice were immunized with β-Gal alone or with indicated adjuvant. 0.2 µg of soluble flagellin and 10^6^ IU of VREP constructs were used. Control mice were given PBS. Each immunized group consisted of five to six mice, and two control mice were used. Serum was assayed for anti-β-Gal IgG, IgG1 and IgG2a by ELISA. A one-way ANOVA with Bonferroni post-hoc test was used to compare the response between mice given sFliC-WT and VREP-FliC-WT, between mice given sFliC-D3 and VREP-FliC-D3, between mice given VREP-FliC-WT or VREP-FliC-D3 and VREP, as well as all adjuvanted groups and mice given β-Gal alone. *P<0.05, **P<0.01 and ***P<0.001.

The deletion mutant FliC-D3 lacks the dominant antigenic hypervariable regions of flagellin while still maintaining adjuvant properties [23]. We compared the adjuvant effect of FliC-WT and FliC-D3 when expressed from VREP. For this purpose, we immunized mice with β-Gal with either VREP-FliC-WT or VREP-FliC-D3 and analyzed their IgG response with ELISA. As controls, we also immunized mice with β-Gal alone or with sFliC-D3, sFliC-WT or VREP-OVA. Both VREP-FliC-WT and VREP-FliC-D3 had an adjuvant effect on the IgG response greater than that of control VREP-OVA or soluble flagellin protein, although VREP-FliC-WT induced slightly higher responses than VREP-FliC-D3 (Fig. 6). In conclusion, VREP expressing either FliC-D3 or FliC-WT has an enhanced adjuvant effect on the IgG response compared to either soluble flagellin or control VREP.

### The Adjuvant Effect of VREP-FliC-D3 is Diminished but not Abrogated in the Absence of Type I IFN Signaling

**Figure 7:**
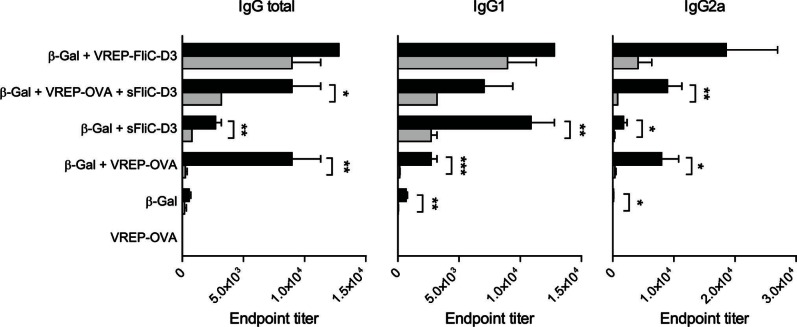
Contribution of type I IFN signaling. *Ifnar1* ^−/−^ (gray bars) and 129sv/ew (black bars) mice were immunized with β-Gal alone or with indicated adjuvant. 0.2 µg of soluble flagellin and 10^6^ IU of VREP constructs were used. Control mice were given VREP-OVA. Each immunized group consisted of five mice, and two control mice were used. Serum was assayed for anti-β-Gal IgG, IgG1 and IgG2a by ELISA. A one-way ANOVA with Bonferroni post-hoc test was used to compare the response between WT and *Ifnar1* ^−/−^ mice given the same vaccination. *P<0.05, **P<0.01 and ***P<0.001.

We previously demonstrated that the adjuvant effect of SFV VREP on the antibody response against co-immunized protein antigen is highly dependent on type I IFNs [43]. We therefore sought to characterize the involvement of type I IFNs on the adjuvant effect exerted by VREP-FliC-D3 or control VREP-OVA with sFliC-D3. For this purpose, we used knockout mice that lack type I IFN signaling (*Ifnar1^−/−^* mice). WT and *Ifnar1^−/−^* mice were immunized with β-Gal alone or with one of the following adjuvants: VREP-FliC-D3, VREP-OVA+sFliC-D3, sFliC-D3 or VREP-OVA. In accordance with previous observations, the adjuvant effect of VREP-OVA was completely abolished in the absence of type I IFN signaling (Fig. 7). The weak response induced by β-Gal alone in WT mice was also greatly diminished in *Ifnar1^−/−^* mice. In groups given sFliC-D3, either alone or with control VREP, the IgG response was decreased but not abolished in *Ifnar1^−/−^* mice. This was mainly due to the IgG2a response, which was abrogated in these groups. These mice produced IgG1 antibodies, although at a lower extent than WT mice. With VREP-FliC-D3, however, an IgG1 response as well as a slight IgG2a response was observed in *Ifnar1^−/−^* mice. Thus, type I IFNs are involved in, but not necessary for, the adjuvant effect of VREP-FliC-D3 on the IgG response.

### IgG Responses are Decreased in the Absence of TLR5 Signaling

**Figure 8:**
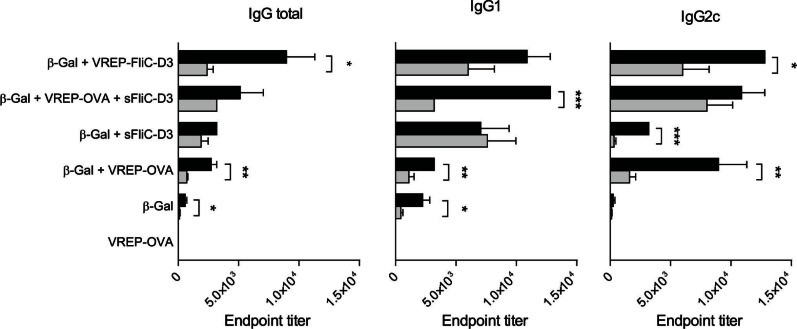
Contribution of TLR5 signaling. *Tlr5* ^−/−^ (gray bars) and C57BL/6N (black bars) mice were immunized with β-Gal alone or with indicated adjuvant. 0.2 µg of soluble flagellin and 10^6^ IU of VREP constructs were used. Control mice were given VREP-OVA. Each immunized group consisted of five (WT) or six (*Tlr5* ^−/−^) mice, and two-three control mice were used. Serum was assayed for anti-β-Gal IgG, IgG1 and IgG2c by ELISA. A one-way ANOVA with Bonferroni post-hoc test was used to compare the response between WT and *Tlr5* ^−/−^ mice given the same vaccination. *P<0.05, **P<0.01 and ***P<0.001.

FliC signals through TLR5, and therefore we examined the role of TLR5 signaling on the adjuvant effect of VREP-FliC-D3 and VREP with sFliC-D3. We assessed the total IgG response as well as the IgG1 response, which are indicative of Th2 type IgG. Because we used mice with a C57BL/6 background in these experiments, we examined the IgG2c response as an indicator of Th1 type IgG. We therefore immunized *Tlr5^−/−^* and WT mice in the same manner as described above for *Ifnar1^−/−^* mice. In accordance with previous results [28], [55], the total IgG response induced by sFliC-D3 decreased only slightly in the absence of TLR5 signaling, and the IgG1 response remained unaffected (Fig. 8). Our results further revealed that the IgG2c response induced by sFliC-D3 is highly dependent on TLR5 signaling. For the VREP adjuvant, both subtypes of IgG were diminished in the absence of TLR5 signaling. When coimmunizing with the VREP and sFliC-D3 adjuvants mixed, the IgG response was slightly diminished. Interestingly, the IgG1 response in *Tlr5^−/−^* mice given VREP and sFliC-D3 was lower than that in mice immunized with the sFliC-D3 adjuvant only, although the IgG2c response remained high in mice given the VREP and FliC-D3 adjuvant. The adjuvant effect of VREP-FliC-D3 was greatly diminished in *Tlr5^−/−^* mice. This was evident on both the IgG1 and IgG2c responses, which were decreased although not abolished. The response in mice given β-Gal without adjuvant was also diminished in the absence of TLR5 signaling.

## Discussion

Here we demonstrate that VREP particles bearing the flagellin gene possess adjuvant properties more potent than that of control VREP particles or soluble flagellin. Co-immunizing VREP-FliC-D3 or VREP-FliC-WT with protein antigen resulted in enhanced antigen-specific antibody responses. VREP-FliC-D3 and VREP-FliC-WT induced antibodies of both the IgG2a and IgG1 isotypes, indicative of both Th1 and Th2 type antibodies. Adding soluble flagellin to control VREP particles, however, did not increase IgG responses compared to administering only VREP particles or soluble flagellin as adjuvant.

The difference in the adjuvant effect exerted by VREP expressing intracellular flagellin compared to VREP administered with soluble flagellin could be due to timing and co-localization of expression. With VREP expressing flagellin, infected cells first amplify VREP RNA and stimulate PRRs such as endosomal TLRs 3, 7 and 8 as well as the cytoplasmic PRR MDA-5, resulting in a strong type I IFN response. At the same time and in the same cell, flagellin is produced, which can then signal through TLR5 and possibly cytoplasmic NLRC4. When VREP is administered with soluble flagellin, however, flagellin is immediately available for PRR stimulation, whereas the VREP effect is exerted after establishing infection and amplifying the viral genome, a lag of several hours. Timing of events as well as co-localization of VREP and flagellin may be crucial for the adjuvant effect of VREP-FliC-WT and VREP-FliC-D3.

In this study, we used VREP encoding flagellin as an adjuvant co-immunized with protein antigen. VREP can also function as a vector encoding the antigen of interest, inducing strong CD8+ T cell and antibody responses [43], [53], [54], [56], [57]. However, we did not observe an increase in cellular or humoral immune responses when co-administering VREP encoding an antigen with VREP encoding flagellin or soluble flagellin. Flagellin has been characterized in several studies as an adjuvant fused with the protein antigen [17], [18], [19], [25], [29], [30], [31], [32]. We therefore also produced VREP encoding flagellin fused with an antigen, but again did not observe an increase in antigen-specific CD8+ T cell or antibody responses compared to VREP encoding the antigen without flagellin. Adding a signal sequence for secretion on the fusion protein also had no effect (data not shown). We therefore focused our present study on increasing the immune response against a soluble protein antigen.

VREP encoding full-length flagellin induced a slightly higher IgG response than the truncated form of flagellin. This was evident on both the IgG1 and IgG2a responses. Previous studies have shown differential systemic and mucosal pro-inflammatory responses to full-length and truncated flagellin, although the mechanism is not clear. When flagellin is administered as soluble protein or incorporated into VLPs, full-length flagellin is more effective in inducing systemic antibody responses while truncated flagellin induces a stronger mucosal antibody response [23], [33], [58]. However, truncated flagellin holds the advantage that it neither induces or is a target of anti-FliC antibody responses that could impair its ability to stimulate TLR5 [23]. Furthermore, expressing truncated flagellin from VREP completely eliminates any induction of anti-FliC antibodies (Fig. S1). Although reports have demonstrated that prior immunity to flagellin does not impair its ability to promote immune responses [59], [60], it is desirable to eliminate any irrelevant immune responses that could potentially lead to harmful effects or attenuate the booster effect of the adjuvant if FliC were to be used in several immunization regimens.

In accordance with previous results [43], we observed that type I IFNs were crucial for the adjuvant effect of VREP on the antibody response against co-immunized protein antigen. For protein antigen co-immunized with flagellin in a soluble form alone or with VREP, or with VREP encoding flagellin, the response was diminished but not abolished in the absence of type I IFN signaling. For groups that were given VREP with soluble flagellin or VREP encoding flagellin, the response was likely diminished due to the lack of contribution from VREP on inducing innate immunity. Mice that were given soluble flagellin without VREP, however, also displayed a decreased antibody response, suggesting that the adjuvant effect of flagellin is also partially dependent on type I IFNs. In *Ifnar1^−/−^* mice given either the sFliC-D3, VREP particles not expressing FliC-D3, or sFliC-D3 with VREP adjuvants, IgG2a antibodies were not induced. VREP-FliC-D3, however, did induce IgG2a antibodies in the absence of type I IFN signaling, indicating that different innate pathways are involved when flagellin is administered as a genetic adjuvant compared to its soluble form.

The role of type I IFNs on the adjuvant effect of flagellin is not fully characterized. In human monocyte-derived dendritic cells, flagellin failed to induce type I IFNs [7], [13], although a direct induction of type I IFNs by FliC-WT has been observed in murine bone marrow-derived macrophages [61]. When flagellin was expressed from the paramyxovirus simian virus 5, production of low levels of IFN-β was observed in epithelial cells *in vitro* [14]. Also, flagellin and IFN-β together, but not by themselves, induces production of T cell chemoattractants in dendritic cells [13]. Flagellin has an adjuvant effect on the CD4+ T cell response [24], a process which involves type I IFN signaling [62]. The lack of proper CD4+ T cell response may explain the diminished IgG responses observed in the lack of type I IFN signaling. However, the observation that the response was not totally abolished illustrates that flagellin also can promote antigen-specific IgG responses in a type I IFN-independent manner.

The adjuvant effect of flagellin has been reported to be codependent on TLR5 and the cytoplasmic PRR NLRC4 [28], although TLR5 alone was necessary for the adjuvant effect of flagellin administered as fusion proteins with the antigen [19]. We demonstrate here that the adjuvant effect of VREP-FliC-D3 is diminished but not abrogated in the absence of TLR5 signaling, indicating that this adjuvant potentiates the IgG response through TLR5 as well as other pathways. It is plausible that flagellin expressed intracellularly from VREP signals through NLRC4, resulting in induction of IL-1β and IL-18, which may account for the adjuvant effect on Th1 type antibodies. Signaling through NLRC4 furthermore causes cell death via pyroptosis [63], which would release flagellin into extracellular space where it can access TLR5 on the surface of cells and thus additionally induce Th2 type antibodies.

Surprisingly, we also observed a decrease in IgG responses in *Tlr5* ^−/−^ mice given protein antigen alone or with the VREP adjuvant. TLR5 is only known to recognize flagellin, and thus we did not expect these groups to be affected. The decrease could be explained by the phenotype of *Tlr5* ^−/−^ mice, which are known to develop spontaneous colitis [64]. Another possible explanation is that flagellin has been shown to induce IFN-β in TLR5-expressing bone marrow-induced macrophages in a TRIF-independent manner [61], suggesting that the lack of TLR5 may have an impact of type I IFN signaling, and thus the formation of IgG in these mice.

In conclusion, we have demonstrated that incorporating flagellin into VREP greatly potentiates antibody responses against an otherwise non-immunogenic protein antigen. Responses were characterized by both Th1 and Th2 type antibodies and were dependent on type I IFN and TLR5 signaling. Thus, where a balanced Th1/Th2 response is likely to be of importance, the use of VREP encoding flagellin may be an attractive choice of adjuvant.

## Supporting Information

Figure S1 **Induction of anti-flagellin antibodies is avoided by incorporation of FliC-D3 in the replicon.** We asked whether there was a difference in the anti-flagellin response induced by soluble flagellin and flagellin expressed from VREP. C57BL/6 mice (*n* = 3) were immunized twice with OVA mixed with one of the following: sFliC-WT (light blue), VREP-LacZ+sFliC-WT (dark blue), VREP-FliC-WT (green), VREP-FliC-D3 (red) or VREP-LacZ (black). 1 µg of soluble flagellin and 10^6^ IU of VREP constructs were used. Serum was assayed for anti-FliC-WT IgG by ELISA. Mice that were given sFliC-WT displayed a strong anti-flagellin response. Expressing FliC-WT from VREP resulted in a sharp decrease in the response. By expressing the truncated FliC-D3 from VREP, induction of anti-flagellin antibodies was completely eliminated.
(EPS)
